# Completely assembled genome sequence of the florfenicol-resistant *Enterococcus faecalis* strain 90_2023 isolated from a raw sausage imported from Italy to Switzerland

**DOI:** 10.1128/MRA.00610-23

**Published:** 2023-09-20

**Authors:** Lucien Kelbert, Marc J. A. Stevens, Jule A. Horlbog, Michael Biggel, Roger Stephan

**Affiliations:** 1 Institute for Food Safety and Hygiene, Vetsuisse Faculty, University of Zürich, Zürich, Switzerland; University of Maryland School of Medicine, Baltimore, Maryland, USA

**Keywords:** *E. faecalis*, florfenicol

## Abstract

Here we report the genome sequence of the florfenicol-resistant *Enterococcus faecalis* strain 90_2023 isolated from a raw-meat sausage (Finocchiona) imported from Italy to Switzerland. It has a genome of 2.75 Mbp and harbors 16 antimicrobial resistance genes, including *cat*A8, *fexA*, and a truncated *optrA* gene on a RepA_N plasmid.

## ANNOUNCEMENT

The use of florfenicol in livestock animals has selected for florfenicol-resistant enterococci harboring resistant determinants including *cat*, *fexA*, and the genes for the ribosomal protection proteins OptrA and PoxtA (1). OptrA, PoxtA, and Cfr, a rRNA methyltransferase, confer resistance to phenicols and also to oxazolidinones, potentially resulting in cross-resistance between florfenicol, used exclusively in animals, and linezolid, used to treat infections in humans (2, 3).

We collected 98 raw-meat sausages on the retail level in Switzerland in March 2023. An amount of 10 g was homogenized in 90 mL brain heart infusion broth with 6% NaCl (Oxoid, Pratteln, Switzerland) and incubated for 18–24 h at 37°C. Enriched samples were streaked on bile esculin azide agar (Merck, Darmstadt, Germany) supplemented with 10 mg/L florfenicol (Sigma-Aldrich, Buchs, Switzerland) and incubated at 37°C for 48 h. Presumptive *Enterococci* colonies were recovered from one sausage (Finocchiona).

For short- and long-read sequencing, DNA from 90_2023 was extracted from a subculture obtained from a single colony grown for 24 h at 37°C on sheep blood agar using the DNeasy Blood and Tissue Kit (Qiagen). Libraries were prepared using the Nextera DNA Flex Library Preparation Kit and sequenced on an Illumina MiniSeq (Illumina, San Diego, CA, USA). The Illumina-sequencing output was two times 770,833 paired reads of 150 bp, corresponding to an 80-fold genome coverage. Short reads were trimmed using fastp 0.23.2 (4) and quality assessed with FastQC v0.11.9 (https://www.bioinformatics.babraham.ac.uk/projects/fastqc/). Long-read sequencing libraries were prepared without size selection or shearing using the SQK-LSK114 kit and sequenced on a FLO-MIN106 cell (Oxford Nanopore Technologies). Basecalling, demultiplexing, and barcode trimming were performed using guppy v6.5.7 (Oxford Nanopore Technologies) and quality assessment with LongQC v1.2.0 (5). The output was 33,953 reads with a total length of 127,522,932 bp (N50: 6,557 bp), corresponding to a 45-fold genome coverage. A hybrid assembly was generated using Unicycler v0.5.0, which includes circularization and rotation at the *dnaA* or *repA* alleles. The genome was annotated using the NCBI Prokaryotic Genome Annotation Pipeline version 6.4 (6). Default parameters were used for all software.

The genome of *Enterococcus faecalis* 90_2023 consists of a 2,748,665 bp chromosome, the 92,098 bp plasmid p90_2023_A, and the 62,024 bp plasmid p90_2023_B. The GC content of the chromosome is 37.6%, and in total, 2,788 genes and 2,716 CDSs were predicted in the genome.

Isolate 90_2023 belongs to *E. faecalis* cgMSLT complex type 3262, as determined using https://cgmlst.org/. The strain is resistant against chloramphenicol but not linezolid according to CLSI criteria (7). Beside three chloramphenicol/florfenicol resistance genes *cat*A8, *fexA*, and *optrA*, the following further putative resistance genes AAC(6*'*), *aad* (6), ANT (6)-Ia, APH(3*'*)-IIIa, *dfrEG*, *efrAB*, *emeA*, *erm*B, *lnuB*, *lsa*AE, SAT-4, *tet*(45), and *tet*(M) were detected by the resistance genes identifier v5.2.1 (8). *OptrA* is localized on p90_2023_B, a plasmid of the RepA_N type, as determined by plasmidfinder (https://cge.food.dtu.dk/services/PlasmidFinder/). Plasmid p90_2023_B is >99.9% homologous to the enterococcal plasmid pAR_0780 (accession number CP063981.1) yet has a 4.9-kbp deletion from directly downstream of a transposase to the *optrA* gene. The deletion truncates the *optrA* gene, leaving only a 133 bp residue.

## Data Availability

Sequencing data were deposited in GenBank and the Sequence Read Archive. The accession numbers of the genome are CP127177, CP127178, and CP127179, and the accession numbers of the reads are SRR24834282 and SRR24834283.
